# Immunogenicity of one and two doses of Gardasil®9 in Tanzanian girls in the DoRIS Trial

**DOI:** 10.1038/s41541-025-01344-1

**Published:** 2025-12-19

**Authors:** Troy J. Kemp, Kathy Baisley, John Changalucha, Jackton Indangasi, Hilary Whitworth, Charles J. Lacey, David Mwanzalima, Ramadhan Hashim, Saidi Kapiga, Richard J. Hayes, Ligia A. Pinto, Deborah Watson-Jones

**Affiliations:** 1 Frederick National Laboratory for Cancer Research, Leidos Biomedical Research Inc, Frederick, MD USA; 2 London School of Hygiene & Tropical Medicine, London, UK; 3 Mwanza Intervention Trials Unit, Mwanza, Tanzania; 4 University of York, York, UK

**Keywords:** Diseases, Immunology, Medical research, Microbiology

## Abstract

Tanzanian girls received one to three doses of Gardasil®9 in the DoRIS randomised trial. Blood samples from one and two-dose Gardasil®9 recipients were collected at months (M)0, M7, M12, M24, and M36 for testing via an HPV Multiplex immunoassay. All participants were HPV 16, HPV 31, and HPV 58 seropositive at M36. For the one-dose group, 96.8–98.2% of girls were seropositive for HPV 11, HPV 18, and HPV 33 and 73.0-87.7% seropositive for HPV 6, HPV 45 and HPV 52. For the two-dose group, all participants were HPV 6, HPV 11, HPV 18, and HPV 33 seropositive, while 98.6% seropositive for HPV 45 and HPV 52. Geometric mean concentrations (GMCs) were higher in the two-dose group compared to the one-dose group but all GMCs in the one-dose group had plateaued by M24 and remained stable to M36. HPV vaccine immune responses in this study will continue through nine years post-vaccination. ClinicalTrials.gov ID: NCT02834637; Registration Date: 2016-06-29; https://www.clinicaltrials.gov/study/NCT02834637.

## Introduction

Prophylactic human papillomavirus (HPV) vaccination is a key component in the World Health Organization (WHO) strategy to accelerate the elimination of cervical cancer as a public health problem by 2030^1^. WHO updated their position in 2022 on HPV vaccination schedules to include an off-label recommendation for one dose of HPV vaccine for 9–20-year-old females and males^2^ after reviewing data from clinical trials and observational studies^3–7^. The KEN SHE randomized controlled trial reported high efficacy against cervical incident persistent HPV 16 and HPV 18 infections up to 36 months after single dose of either a bivalent (Cervarix®; 2vHPV, 97.5% efficacy) or a nonavalent (Gardasil®9; 9vHPV, 98.8% efficacy) vaccine in Kenyan females^8^. In addition, observational studies of one-dose recipients in Costa Rica and India have shown sustained protection against persistent HPV 16 and HPV 18 infection for up to 11 years for Cervarix® and 12 years for Gardasil®, respectively^5,9^. Those studies also demonstrated durable and high HPV 16 and HPV 18 seropositivity up to 16 years for Cervarix® and 10 years for Gardasil®^10,11^. Immunogenicity results from the DoRIS randomised trial in Tanzania, which measured antibody responses in one-, two-, and three-dose recipients of either the bivalent (Cervarix®; 2vHPV) or nonavalent (Gardasil®9; 9vHPV) vaccines showed sustained and high HPV 16 and HPV 18 seropositivity after one dose up to Month (M)60^12^. An immunobridging study found that one-dose IgG antibody responses to HPV 16 and HPV 18 in the DoRIS study at M24 were non-inferior to those of single dose recipients in the KEN SHE trial where efficacy for a single dose of Cervarix® and Gardasil®9 had been demonstrated^13^, which starts to provide insight into antibody levels that offer protection.

To date, most of the one-dose efficacy and immunogenicity results have focused on HPV 16 and HPV 18 as these two genotypes cause 70% of cervical cancer cases worldwide; though, other high-risk genotypes such as HPV 31, 33, 45, 52, and 58 also cumulatively cause ~18% of cervical cancer cases worldwide, while the low-risk genotypes HPV 6 and HPV 11 cause 90% of genital warts cases^14,15^. The aim of this study was to evaluate the seropositivity, levels and durability of antibody responses to the vaccine related HPV types in Gardasil®9 recipients across a single dose group and a two-dose group as this coincides with the current vaccine label indications (two doses of HPV vaccine for 9–14 year old participants who are immunocompetent) and the off-label (single dose of HPV vaccine for 9–20 year old participants who are immunocompetent) recommendation from WHO^2^. Here, we describe the results from a subset of DoRIS trial Gardasil®9 one-dose and two-dose recipients on the seropositivity, levels and durability of antibody responses to all 9 HPV genotypes included in this vaccine up to M36, using a 9-plex Luminex-based assay.

## Results

### Characteristics of the participants

Baseline characteristics (including baseline HPV DNA positivity and antibody positivity) of the random sub-sample of 150 participants (75 per arm) were similar between arms (Table 1). Consistent with random sampling, characteristics of the participants in the sub-sample were similar to those who were not sampled (Supplementary Table 1).

**Table 1:** Baseline characteristics of random sample of DoRIS trial participants in one-dose and two-dose Gardasil®9 arms selected for multiplex immunoassay testing

	1D Gardasil®9	2D Gardasil®9	Total
	***N*** = **75**	***N*** = **75**	***N*** = **150**
Median (IQR) age (years)	10 (9-12)	11 (10-12)	10 (9-12)
Age group			
9–10 years	44 (58.7%)	34 (45.3%)	78 (52.0%)
11–12 years	18 (24.0%)	25 (33.3%)	43 (28.7%)
13–14 years	13 (17.3%)	16 (21.3%)	29 (19.3%)
School type			
Primary	63 (84.0%)	61 (81.3%)	124 (82.7%)
Secondary	12 (16.0%)	14 (18.7%)	26 (17.3%)
Passed menarche			
Yes	9 (12.0%)	9 (12.0%)	18 (12.0%)
Ever cleansed vagina			
Yes	6 (8.0%)	5 (6.7%)	11 (7.3%)
Ever had vaginal sex			
Yes	1 (1.3%)	3 (4.0%)	4 (2.7%)
HPV DNA positive^a^			
HPV 6	1 (1.3%)	0 (0.0%)	1 (0.7%)
HPV 11	0 (0.0%)	0 (0.0%)	0 (0.0%)
HPV 16	0 (0.0%)	0 (0.0%)	0 (0.0%)
HPV 18	0 (0.0%)	0 (0.0%)	0 (0.0%)
HPV 31	0 (0.0%)	0 (0.0%)	0 (0.0%)
HPV 33	0 (0.0%)	0 (0.0%)	0 (0.0%)
HPV 45	0 (0.0%)	0 (0.0%)	0 (0.0%)
HPV 52	0 (0.0%)	0 (0.0%)	0 (0.0%)
HPV 58	0 (0.0%)	0 (0.0%)	0 (0.0%)
Any 9-valent HPV type DNA positive^a^	1 (1.3%)	0 (0.0%)	1 (0.7%)
HPV antibody seropositive^a^			
HPV 6	19 (25.3%)	14 (18.7%)	33 (22.0%)
HPV 11	20 (26.7%)	10 (13.3%)	30 (20.0%)
HPV 16	2 (2.7%)	0 (0.0%)	2 (1.3%)
HPV 18	1 (1.3%)	1 (1.3%)	2 (1.3%)
HPV 31	1 (1.3%)	2 (2.7%)	3 (2.0%)
HPV 33	12 (16.0%)	13 (17.3%)	25 (16.7%)
HPV 45	2 (2.7%)	1 (1.3%)	3 (2.0%)
HPV 52	1 (1.3%)	0 (0.0%)	1 (0.7%)
HPV 58	4 (5.3%)	5 (6.7%)	9 (6.0%)
Any 9-valent HPV type seropositive^a^	33 (44.0%)	32 (42.7%)	65 (43.3%)

^a^DoRIS participant’s status at baseline (pre-vaccination).

Serology results were available at all timepoints for all 150 participants. The per-protocol cohort ranged from 55/75 participants (73.3%; HPV 11) to 74/75 (98.7%; HPV 18, HPV 31, and HPV 52) in the one-dose arm, and from 60/75 participants (80.0%; HPV 6) to 74/75 (98.7%; HPV 16 and HPV 52) in the two-dose arm.

### Seropositivity of participants in one- and two-dose arms

In the one-dose arm, all participants in the per-protocol cohort were seropositive for HPV 16, HPV 31, and HPV 58 at M24 and M36, and >95% were seropositive for HPV 11, HPV 18, and HPV 33 (Table 2). For the other three genotypes (HPV 6, HPV 45, and HPV 52), the percentage of one-dose participants in the per-protocol cohort who were seropositive ranged from 70.3% (HPV 52, M24) to 87.7% (HPV 45, M36) (Table 2) for M24 and M36.

**Table 2:** Comparisons of antibody seropositivity at M7 to M36 after one- or two-doses of Gardasil®9 in DoRIS trial (per-protocol cohort^a^)

	One-dose		Two-doses		Difference in seropositivity^b^ (exact 95% CI)
	*N*	Seropositive^b^ (%)	N	Seropositive^b^ (%)	[One-dose] – [Two-dose]
**Gardasil®9**					
Month 7					
HPV 6	56	46 (82.1%)	60	60 (100%)	–17.9% (–30.4, –8.8)
HPV 11	55	54 (98.2%)	64	64 (100%)	–1.8% (–9.8, 4.3)
HPV 16	73	73 (100%)	74	74 (100%)	0
HPV 18	74	74 (100%)	73	73 (100%)	0
HPV 31	74	73 (98.6%)	72	72 (100%)	–1.4% (–7.3, 3.9)
HPV 33	63	63 (100%)	61	61 (100%)	0
HPV 45	73	60 (82.2%)	73	73 (100%)	–17.8% (–28.7, –9.8)
HPV 52	74	60 (81.1%)	74	74 (100%)	–18.9% (–29.7, –10.7)
HPV 58	71	70 (98.6%)	69	69 (100%)	–1.4% (–7.6, 4.2)
Month 12					
HPV 6	56	44 (78.6%)	60	60 (100%)	–21.4% (–34.4, –11.6)
HPV 11	55	54 (98.2%)	64	64 (100%)	–1.8% (–9.8, 4.3)
HPV 16	73	73 (100%)	74	74 (100%)	0
HPV 18	74	72 (97.3%)	73	73 (100%)	–2.7% (–9.5, 2.6)
HPV 31	74	73 (98.6%)	72	72 (100%)	–1.4% (–7.3, 3.9)
HPV 33	63	62 (98.4%)	61	61 (100%)	–1.6% (–8.8, 4.5)
HPV 45	73	59 (80.8%)	73	73 (100%)	–19.2% (–30.1, –10.9)
HPV 52	74	51 (68.9%)	74	74 (100%)	–31.1% (–42.9, –20.8)
HPV 58	71	69 (97.2%)	69	69 (100%)	–2.8% (–10.1, 2.9)
Month 24					
HPV 6	56	46 (82.1%)	60	60 (100%)	–17.9% (–30.4, –8.8)
HPV 11	55	53 (96.4%)	64	64 (100%)	–3.6% (–12.7, 2.6)
HPV 16	73	73 (100%)	74	74 (100%)	0
HPV 18	74	71 (95.9%)	73	73 (100%)	–4.1% (–11.5, 1.2)
HPV 31	74	74 (100%)	72	72 (100%)	0
HPV 33	63	62 (98.4%)	61	61 (100%)	–1.6% (–8.8, 4.5)
HPV 45	73	63 (86.3%)	73	72 (98.6%)	–12.3% (–22.4, –4.0)
HPV 52	74	52 (70.3%)	74	74 (100%)	–29.7% (–41.5, –19.7)
HPV 58	71	71 (100%)	69	69 (100%)	0
Month 36					
HPV 6	56	45 (80.4%)	60	60 (100%)	–19.6% (–32.4, –10.2)
HPV 11	55	54 (98.2%)	64	64 (100%)	–1.8% (–9.8, 4.3)
HPV 16	73	73 (100%)	74	74 (100%)	0
HPV 18	74	72 (97.3%)	73	73 (100%)	–2.7% (–9.5, 2.6)
HPV 31	74	74 (100%)	72	72 (100%)	0
HPV 33	63	61 (96.8%)	61	61 (100%)	–3.2% (–11.1, 3.0)
HPV 45	73	64 (87.7%)	73	72 (98.6%)	–11.0% (–20.8, –2.9)
HPV 52	74	54 (73.0%)	74	73 (98.6%)	–25.7% (–37.5, –15.1)
HPV 58	71	71 (100%)	69	69 (100%)	0

^a^DoRIS participants who were antibody negative and DNA negative at baseline (pre-vaccination) for the HPV genotype under analysis.

^b^Seropositivity defined as antibody concentrations above the laboratory determined cut-off.

In the two-dose arm, all participants in the per-protocol cohort were seropositive at M24 for each of the HPV genotypes in the nonavalent vaccine except for HPV 45, where 98.6% were seropositive (Table 2). At M36, all participants were seropositive for 7 of the 9 vaccine-related genotypes, and 98.6% were seropositive for HPV 45 and HPV 52. The difference in seropositivity between the one-dose and two-dose arms was greatest for HPV 52 (M24, –29.7%; M36, –25.7%), followed by HPV 6 (M24, –17.9%; M36, –19.6%) and HPV 45 (M24, –12.3%; M36, –11.0%).

### Longitudinal antibody responses of participants in one- and two-dose arms

**Fig. 1:**
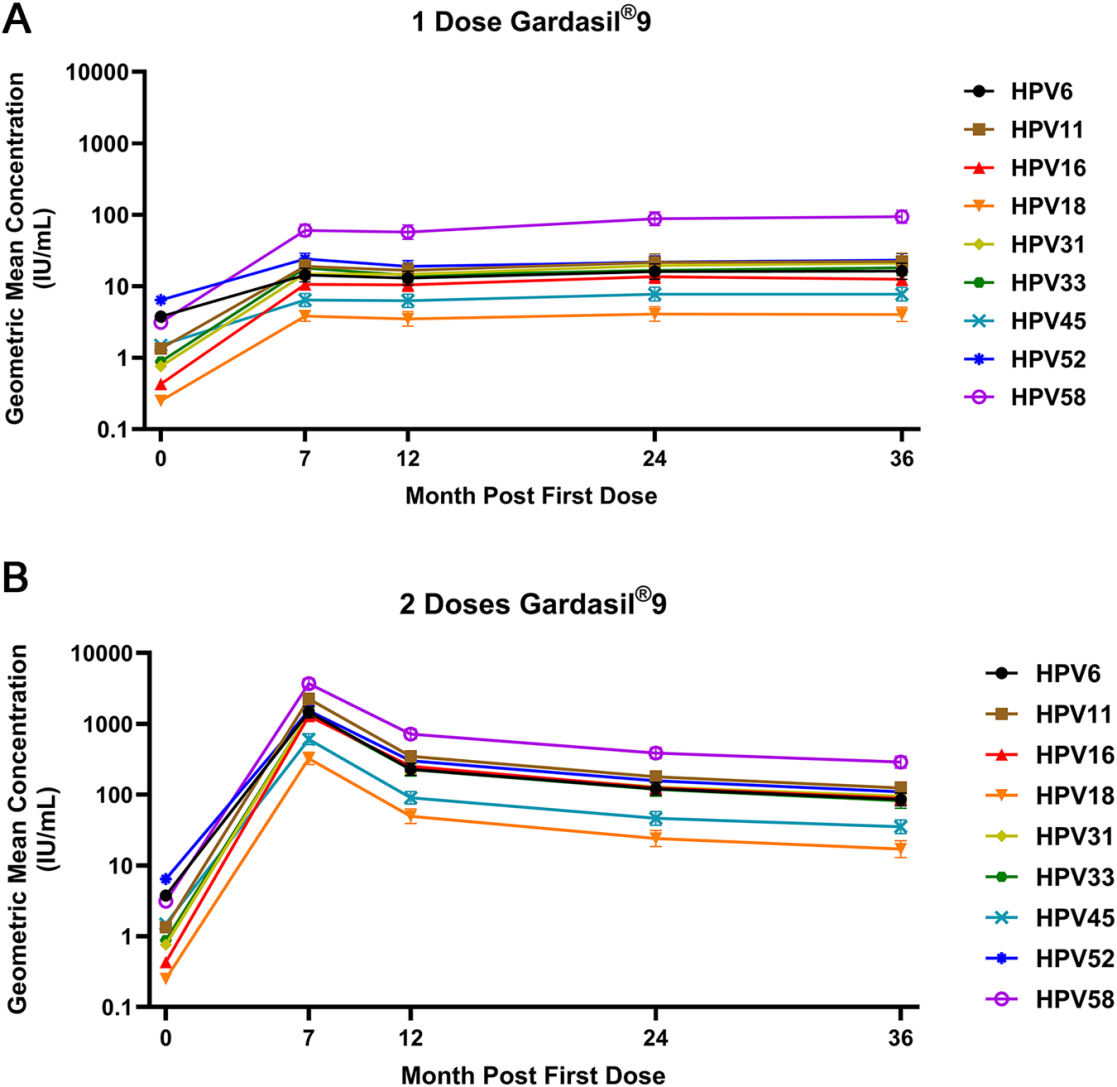
Stability of HPV IgG antibody levels up to Month (M)36 in the per-protocol cohort amongst one- and two-dose recipients of the 9vHPV vaccine. **A** Depicts the geometric mean concentration in International Units (IU) per milliliter (mL) anti-HPV IgG antibody responses for the nine HPV genotypes included in the 9vHPV vaccine for one-dose recipients of the vaccine, while **B** depicts the geometric mean concentration in IU/mL anti-HPV IgG antibody responses for the nine HPV genotypes included in the 9vHPV vaccine for two-dose recipients of the vaccine. The x-axis describes the timepoints (M0, M7, M12, M24, and M36) of sample testing, and the y-axis indicates the measured concentration in IU/mL. The symbols indicate the geometric mean concentration (IU/mL) for each HPV genotype evaluated, and the error bars associated with each symbol indicate the 95% confidence interval.

In the one-dose arm, anti-HPV IgG antibody GMCs reached a plateau around M24, for all 9 HPV genotypes (Fig. 1A). There was no evidence of a difference in antibody GMCs between M36 and M24 for any of the 9 HPV genotypes, with GMC ratios ranging from 0.92 (95% CI, 0.81–1.04; HPV 16) to 1.09 (95% CI, 0.93–1.27; HPV 33) (Table 3). In contrast, antibody GMCs of all nine genotypes were significantly higher at M36 than at M12, with GMC ratios from 1.16 (95% CI, 1.02–1.31; HPV 18) to 1.64 (95% CI, 1.44–1.88; HPV 58).

**Table 3:** Stability of geometric mean concentrations (GMC) between M7 and M36 in DoRIS trial (per-protocol cohort^a^)

	One-dose		Two-doses		GMC ratio^*c*^ (Two-doses/One-dose) 95% CI
	*N*^a^	GMC^b^ (95% CI) (IU/mL)	*N*^a^	GMC^b^ (95% CI) (IU/mL)	
**Gardasil®9**					
**HPV 6**					
Month 7	56	14.3 (11.5, 17.8)	60	1458.8 (1213.0, 1754.4)	101.9 (75.3, 137.8)
Month 12	56	13.1 (10.4, 16.4)	60	227.7 (187.9, 276.0)	17.4 (12.9, 23.6)
Month 24	56	16.1 (12.6, 20.6)	60	121.3 (98.4, 149.6)	7.5 (5.6, 10.2)
Month 36	56	16.3 (12.6, 21.1)	60	85.3 (69.4, 104.9)	5.2 (3.9, 7.1)
*GMC ratio*^*c*^ *(M36 /M7) (95% CI)*		*1.14* (*1.00, 1.29)*		*0.06* (*0.05, 0.07)*	
*GMC ratio*^*c*^ *(M36 /M12) (95% CI)*		*1.25* (*1.10, 1.42)*		*0.37* (*0.33, 0.42)*	
*GMC ratio*^*c*^ *(M36 /M24) (95% CI)*		*1.01* (*0.89, 1.15)*		*0.70* (*0.62, 0.80)*	
**HPV 11**					
Month 7	55	18.9 (15.3, 23.4)	64	2256.9 (1897.5, 2684.5)	119.4 (88.7, 160.7)
Month 12	55	16.6 (13.1, 21.0)	64	347.4 (291.5, 414.1)	20.9 (15.5, 28.1)
Month 24	55	21.5 (16.4, 28.3)	64	177.9 (147.0, 215.3)	8.3 (6.1, 11.1)
Month 36	55	22.2 (17.0, 29.0)	64	124.1 (102.4, 150.5)	5.6 (4.2, 7.5)
*GMC ratio*^*c*^ *(M36 /M7) (95% CI)*		*1.17* (*1.02, 1.35)*		*0.06* (*0.05, 0.06)*	
*GMC ratio*^*c*^ *(M36 /M12) (95% CI)*		*1.33* (*1.16, 1.53)*		*0.36* (*0.31, 0.41)*	
*GMC ratio*^*c*^ *(M36 /M24) (95% CI)*		*1.03* (*0.90, 1.18)*		*0.70* (*0.61, 0.79)*	
**HPV 16**					
Month 7	73	10.6 (8.9, 12.7)	74	1286.6 (1099.0, 1506.2)	121.0 (92.6, 158.1)
Month 12	73	10.5 (8.5, 13.0)	74	250.4 (209.9, 298.7)	23.8 (18.3, 31.1)
Month 24	73	13.7 (11.1, 16.9)	74	124.7 (102.7, 151.3)	9.1 (7.0, 11.9)
Month 36	73	12.5 (10.2, 15.3)	74	88.5 (72.5, 108.2)	7.1 (5.4, 9.2)
*GMC ratio*^*c*^ *(M36 /M7) (95% CI)*		*1.18* (*1.04, 1.33)*		*0.07* (*0.06, 0.08)*	
*GMC ratio*^*c*^ *(M36 /M12) (95% CI)*		*1.19* (*1.05, 1.35)*		*0.35* (*0.31, 0.40)*	
*GMC ratio*^*c*^ *(M36 /M24) (95% CI)*		*0.92* (*0.81, 1.04)*		*0.71* (*0.63, 0.80)*	
**HPV 18**					
Month 7	74	3.8 (3.3, 4.5)	73	324.1 (264.7, 396.7)	84.6 (61.3, 116.7)
Month 12	74	3.5 (2.8, 4.4)	73	49.5 (39.0, 62.7)	14.2 (10.3, 19.5)
Month 24	74	4.1 (3.2, 5.2)	73	24.0 (18.4, 31.1)	5.9 (4.2, 8.1)
Month 36	74	4.0 (3.2, 5.1)	73	17.0 (13.0, 22.4)	4.2 (3.1, 5.8)
*GMC ratio*^*c*^ *(M36 /M7) (95% CI)*		*1.06* (*0.93, 1.20)*		*0.05* (*0.05, 0.06)*	
*GMC ratio*^*c*^ *(M36 /M12) (95% CI)*		*1.16* (*1.02, 1.31)*		*0.34* (*0.30, 0.39)*	
*GMC ratio*^*c*^ *(M36 /M24) (95% CI)*		*0.99* (*0.87, 1.12)*		*0.71* (*0.63, 0.81)*	
**HPV 31**					
Month 7	74	14.9 (12.2, 18.2)	72	1385.8 (1178.5, 1629.5)	92.8 (70.3, 122.6)
Month 12	74	14.7 (12.0, 18.1)	72	236.0 (197.3, 282.4)	16.0 (12.1, 21.2)
Month 24	74	19.5 (15.9, 24.1)	72	128.2 (104.9, 156.8)	6.6 (5.0, 8.7)
Month 36	74	21.1 (16.8, 26.5)	72	94.4 (76.8, 116.0)	4.5 (3.4, 5.9)
*GMC ratio*^*c*^ *(M36 /M7) (95% CI)*		*1.42* (*1.23, 1.62)*		*0.07* (*0.06, 0.08)*	
*GMC ratio*^*c*^ *(M36 /M12) (95% CI)*		*1.44* (*1.25, 1.65)*		*0.40* (*0.35, 0.46)*	
*GMC ratio*^*c*^ *(M36 /M24) (95% CI)*		*1.08* (*0.94, 1.24)*		*0.74* (*0.64, 0.85)*	
**HPV 33**					
Month 7	63	18.0 (14.8, 22.0)	61	1368.4 (1134.8, 1650.1)	75.9 (55.5, 103.8)
Month 12	63	14.2 (11.2, 18.0)	61	225.8 (182.9, 278.9)	15.9 (11.6, 21.7)
Month 24	63	16.7 (13.0, 21.4)	61	119.0 (94.4, 150.1)	7.1 (5.2, 9.8)
Month 36	63	18.1 (14.2, 23.2)	61	81.6 (64.2, 103.6)	4.5 (3.3, 6.2)
*GMC ratio*^*c*^ *(M36 /M7) (95% CI)*		*1.01* (*0.86, 1.17)*		*0.06* (*0.05, 0.07)*	
*GMC ratio*^*c*^ *(M36 /M12) (95% CI)*		*1.27* (*1.09, 1.49)*		*0.36* (*0.31, 0.42)*	
*GMC ratio*^*c*^ *(M36 /M24) (95% CI)*		*1.09* (*0.93, 1.27)*		*0.69* (*0.59, 0.80)*	
**HPV 45**					
Month 7	73	6.4 (5.2, 7.9)	73	605.3 (507.3, 722.2)	94.1 (70.2, 126.2)
Month 12	73	6.3 (5.1, 7.7)	73	90.2 (73.8, 110.2)	14.4 (10.8, 19.3)
Month 24	73	7.8 (6.2, 9.7)	73	46.3 (37.0, 57.9)	6.0 (4.5, 8.0)
Month 36	73	7.8 (6.2, 9.7)	73	35.2 (28.3, 43.8)	4.5 (3.4, 6.1)
*GMC ratio*^*c*^ *(M36 /M7) (95% CI)*		*1.21* (*1.07, 1.36)*		*0.06* (*0.05, 0.07)*	
*GMC ratio*^*c*^ *(M36 /M12) (95% CI)*		*1.24* (*1.10, 1.40)*		*0.39* (*0.35, 0.44)*	
*GMC ratio*^*c*^ *(M36 /M24) (95% CI)*		*1.00* (*0.89, 1.13)*		*0.76* (*0.67, 0.86)*	
**HPV 52**					
Month 7	74	24.1 (20.1, 28.8)	74	1537.6 (1312.7, 1800.9)	63.9 (49.0, 83.3)
Month 12	74	19.0 (15.7, 22.9)	74	300.9 (251.6, 359.9)	15.9 (12.2, 20.7)
Month 24	74	21.7 (17.6, 26.7)	74	156.3 (128.5, 190.0)	7.2 (5.5, 9.4)
Month 36	74	23.2 (18.8, 28.7)	74	110.0 (89.8, 134.8)	4.7 (3.6, 6.2)
*GMC ratio*^*c*^ *(M36 /M7) (95% CI)*		*0.97* (*0.86, 1.08)*		*0.07* (*0.06, 0.08)*	
*GMC ratio*^*c*^ *(M36 /M12) (95% CI)*		*1.23* (*1.09, 1.38)*		*0.37* (*0.33, 0.41)*	
*GMC ratio*^*c*^ *(M36 /M24) (95% CI)*		*1.07* (*0.95, 1.20)*		*0.70* (*0.63, 0.79)*	
**HPV 58**					
Month 7	71	60.4 (49.9, 73.2)	69	3693.5 (3147.1, 4334.8)	61.1 (46.6, 80.2)
Month 12	71	57.1 (45.2, 72.0)	69	717.9 (606.6, 849.6)	12.6 (9.6, 16.5)
Month 24	71	88.0 (70.7, 109.5)	69	386.3 (323.2, 461.6)	4.4 (3.3, 5.8)
Month 36	71	93.8 (76.3, 115.4)	69	287.1 (237.3, 347.4)	3.1 (2.3, 4.0)
*GMC ratio*^*c*^ *(M36 /M7) (95% CI)*		*1.55* (*1.36, 1.78)*		*0.08* (*0.07, 0.09)*	
*GMC ratio*^*c*^ *(M36 /M12) (95% CI)*		*1.64* (*1.44, 1.88)*		*0.40* (*0.35, 0.46)*	
*GMC ratio*^*c*^ *(M36 /M24) (95% CI)*		*1.07* (*0.93, 1.22)*		*0.74* (*0.65, 0.85)*	

^a^DoRIS participants who were antibody negative and DNA negative at baseline (pre-vaccination) for the HPV genotype under analysis.

^b^Serum antibody geometric mean concentration (GMC).

^c^Estimated with linear mixed effect model with log antibody concentration as the response and dose group, time point, and a dose group, time interaction term as fixed effects, and participant as a random effect to account for correlation of repeated measurements within participant.

In the two-dose arm, anti-HPV IgG antibody GMCs peaked at M7 and then slowly declined thereafter (Fig. 1B). Antibody GMCs at M36 were around 30% lower than at M24 and 60% lower than at M12, for all 9 HPV genotypes (Table 3). The decline in antibody GMCs after the peak at M7 was similar for all HPV genotypes, with the greatest decrease between M7 and M12.

As expected, antibody GMCs for all HPV genotypes were significantly higher in the two-dose arm than in the one-dose arm, at all timepoints. At M7, antibody GMCs in the two-dose arm were 61.1 (HPV 58) to 121.0 (HPV 16) times higher than in the one-dose arm (Table 3; Fig. 1A, B). By M36, the fold difference between the arms had greatly reduced, but antibody GMCs in the two-dose arm were still 3.1 (HPV 58) to 7.1 (HPV 16) times higher than in the one-dose arm.

Antibody responses and seropositivity among the total vaccinated cohort were similar to those in the per-protocol cohort, for all HPV genotypes (Supplementary Tables 1 and 2; Supplementary Fig. 1).

## Discussion

As of May 2025, 75 countries had implemented the single dose HPV vaccination schedule, based on the 2022 WHO guidance for the use of a single dose in 9–20 year-old males and females^16^. However, published data on immune responses after a single dose primarily focus on HPV 16 and HPV 18. Our study confirms that a single dose of 9vHPV vaccine induces antibody responses not only for HPV 16 and HPV 18, but also for the other seven genotypes included in the 9vHPV vaccine. Moreover, the antibody responses of the other 7 genotypes follow a similar anti-HPV 16 and HPV 18 kinetic pattern as shown in other studies^10,11^, reaching a plateau at M24 and remaining stable to M36, and with high sustained seropositivity over time.

The one-dose recipients from this study had a similar antibody response profile for HPV 16 and HPV 18 as described in the CVT and India observational studies. The antibody responses in these three studies (DoRIS, CVT, and INDIA) for HPV 16 and HPV 18 plateau around M24 and continue with a stable and durable antibody response^5,10,12^. However, there is very limited immunogenicity data on the other high-risk and low-risk HPV genotypes included in the 9vHPV vaccine amongst one-dose recipients, and here we report that the antibody response profile for each of the 9 HPV genotypes was similar. To note, there are three other studies that describe single dose immunogenicity responses to the 9 HPV genotypes included in the 9vHPV vaccine. Two of the studies (Bornstein et al.^17^ and Klein et al.^18^) were tested with MSD’s competitive Luminex Immunoassay (cLIA) and/or immunoglobulin G-Luminex immunoassay (IgG-LIA). The third study (Sahasrabuddhe et al.^19^) was tested with the LIA9v assay presented here as well as a pseudovirion-based neutralization assay (PBNA). The Bornstein et al. study evaluated the antibody response and seropositivity to the 9 HPV genotypes only up to 12 months following the first dose. Unfortunately, this study did not capture the antibody responses amongst the single dose participants out to 36 months as described in the present study because the study design limited this assessment. Furthermore, the seropositivity assessment at M12 with the cLIA ranged from 31.1% to 93.2%, while the seropositivity assessment at M12 with the IgG-LIA ranged from 57.9% to 98.8%^17^, which the IgG-LIA results were closer to the results we reported here (68.9%–100% seropositivity). In another study described by Sahasrabuddhe et al., which was conducted in the USA, was only able to report single dose immunogenicity results out to month 24 due to study design limitations. The seropositivity range at M24 post first dose of the 9vHPV vaccine was 74%–100% (LIA9v assay) and 82%–100% (Pseudovirion-based neutralization assay) for the 9vHPV vaccine-related genotypes amongst 9–11 year old participants. These seropositivity ranges were similar to what we reported here at M24 (70.3%–100%, LIA9v). Lastly, the Klein et al. study describes the immunogenicity for the 9vHPV vaccine-related genotypes amongst 10–15 year old participants who did not receive their second dose of 9vHPV vaccine until 12–54 months after their first dose^18^. The seropositivity, based on MSD’s cLIA, of these participants ranged from 28.1% to 70.2% in the 12–24 Month Dose Interval Group (DIG), 22.5%–54.9% in the 24–36 Month Dose Interval Group (DIG), and 38.9%-72.2% in the 36–54 Month Dose Interval Group (DIG), which is much lower than the seropositivity range described in this study from M12-M36 (Table 2). The difference in seropositivity between the two studies is most likely associated with the assay utilized to test the respective study along with the cutoff established for each assay. The cLIA measures the competition for antibody binding to a single neutralizing epitope of each HPV type-specific VLP as opposed to the IgG-LIA^20^ and LIA9v, which measure HPV IgG type-specific antibodies regardless of neutralizing potential. As described in the Bornstein et al. study, the IgG-LIA had higher seropositivity as compared to the cLIA for all genotypes except HPV 58^17^, and the IgG-LIA is a similar assay to the LIA9v described here for the DoRIS study.

Though seropositivity is high for all nine 9vHPV vaccine-related genotypes in the one-dose arm, we observed that some participants were not seropositive at M7. This is especially noted for HPV 6, HPV 45, and HPV 52. We did not test the M1 samples from these participants to determine if there was a loss of seropositivity, or if the participants never seroconverted. However, some of the participants who were seronegative at M7 were seropositive at later visits, which suggests that the antibody responses from some of the participants were at the cutoff of the assay. A reduction in seropositivity amongst single dose participants receiving a 9vHPV vaccine was also described in the Bornstein et al. and Klein et al. studies. While the Bornstein et al. study evaluated seropositivity at one month following vaccination, the Klein et al. study only evaluated seropositivity at least one year post vaccination, so the lack of seroconversion can only be evaluated in the Bornstein et al. study, which showed a seropositivity range of 56% (HPV 45, girls) to 97.9% (HPV 11, girls) at 1 month post one dose of 9vHPV^17^. Other studies also noted the decrease in seropositivity amongst participants who received only one dose of HPV vaccine (4vHPV^21^ or 2vHPV^22^); however, one-dose recipients in Costa Rica and India have shown sustained protection against persistent HPV 16 and HPV 18 infection for up to 11 years for 2vHPV and 12 years for 4vHPV, respectively^5,9^. In addition, related to the 9vHPV vaccine, the vaccine efficacy remains very high (95.5%) for the 9vHPV vaccine related HPV types as noted with the KEN SHE trial^8^. The lack of 100% agreement between the serology assay seropositivity results and vaccine efficacy may suggest three possible explanations that may complement each other or be independent: 1) exposure to HPV in the genital tract may activate local memory B cells to produce sufficient quantities of neutralizing antibodies to maintain protection, 2) additional optimization of the immunoassays in relation to sensitivity and assay cutoff for population based factors, or 3) genetics, where the immune response to a single dose of HPV vaccine may induce slightly different levels of antibodies, from undetectable to detectable in different populations. To further understand the long-term stability of the antibody levels and seropositivity, the DoRIS trial will continue to follow their one-dose and two-dose participants for nine years. The trial is currently in its eighth year of follow-up, with 90% retention. The DoRIS trial was not powered to look at efficacy of virological endpoints; however, girls will be encouraged to attend for cervical screening through the national screening program when eligible.

When assessing the plateau phase of the antibody responses in the one dose arm, we observed that the antibodies for each of the 9 HPV genotypes reached a plateau by M24 and remained stable up to M36. We also note that the antibody GMC ratios between M12 and M36 indicate a slight increase in antibody levels; however, the GMC ratios stabilize between M24 to M36. The slight increase in antibody levels between M12 and M36 in the one dose arms is unlikely due to newly acquired HPV infections, since this phenomenon was observed with all nine HPV types. Furthermore, only 4 participants in the one dose arm reported ever having had sex by M36. We also do not suspect it is assay related as longitudinal collections were tested sequentially in the assay, within the same experimental batch.

In an effort to standardize our reporting of HPV immunogenicity results, we performed our testing with an assay that was calibrated to the nine individual WHO anti-HPV International Standard antibodies (anti-HPV 6, 11, 16, 18, 31, 33, 45, 52, and 58), which were developed to harmonize data reporting amongst HPV clinical trials. Further use of the HPV International Standard antibodies by more laboratories will allow for better comparisons of immunogenicity data between trials to better inform public health policy makers on vaccine development and dosing regimen strategies.

A limitation of this study is that HPV antibody responses were only measured up to M36; however, the DoRIS study will continue to collect serum from these participants up to M108, and the durability of the anti-HPV antibody responses will be evaluated for the 9 HPV genotypes at these later time points. Furthermore, it will be important to continue immunobridging the DoRIS study to the KEN SHE study to assess the linkage between immunogenicity and protection for the 9 HPV genotypes at later time points, especially considering that the potential for exposure to high-risk HPV genotype infections for these participants to increase after M36. This study did not test samples from the 2vHPV vaccine recipients with the multiplex immunoassay. It will be important to understand the antibody responses elicited by the 2vHPV vaccine (both one-dose and two-dose participants) to cross-protected genotypes HPV 31, 33, and 45. Although the reported vaccine efficacy in exploratory analysis for a single dose of 2vHPV vaccine was 10.1% for HPV 31, 33, and 45 at M36 within the KEN SHE trial^8^, it will be important to understand this impact at later time points as well as based on individual HPV types. Another limitation of this study relates to the generalizability of this data as it was only conducted in a single country and in female participants. It will be important to evaluate these parameters in other countries across the world as well as include male participants to understand if the antibody responses are affected by these variables. Studies are underway in Tanzania to address these questions in males.

In conclusion, the anti-HPV IgG antibody responses for the nine HPV genotypes included in the 9vHPV vaccine were stable and durable up to M36 amongst the one-dose recipients in the primary target age for vaccination. Furthermore, the seropositivity at M36 was high for each of the vaccine-related HPV genotypes amongst the one-dose recipients. These results, along with the reported efficacy of 95.5% (modified intention-to-treat cohort; HPV 16, 18, 31, 33, 45, 52, 58)^8^ amongst one-dose recipients of the 9vHPV vaccine in the KEN SHE trial suggests the 9vHPV vaccine will offer sustained immune responses and protection when given to girls in the current primary target age range for vaccination. As the DoRIS trial will continue to monitor the recipients of one-dose or two-dose 9vHPV vaccine up to nine years, we will obtain further data on the long-term durability of the HPV antibody response to all 9 HPV genotypes included in the nonavalent vaccine.

## Methods

### Study design

A randomised, unblinded, phase 3 trial of two HPV vaccines was conducted in Mwanza, northwestern Tanzania (Dose Reduction Immunobridging and Safety Study of two HPV vaccines in Tanzanian girls [DoRIS]). The study was registered under clinicaltrials.gov (NCT02834637; Registration Date: 2016-06-29) and was approved by the Tanzanian Medical Research Coordinating Committee and the ethics committee of the London School of Hygiene & Tropical Medicine. Regulatory approval was given by the Tanzanian Medicines and Medical Devices Authority (TMDA).

### Participants

The trial protocol and procedures have been described previously^23^. Briefly, 930 girls living in Mwanza, Tanzania aged 9–14 years, and human immunodeficiency virus (HIV)-negative at screening, were enrolled into the study. Each participant provided assent following written informed consent from parents or guardians. Participants were randomized to one of six arms (*N* = 155 participants per arm) comprising three different dose schedules of two different HPV vaccines (bivalent vaccine [2vHPV], Cervarix®, GSK Biologicals, or nonavalent vaccine [9vHPV], Gardasil®9, MSD). The three dose schedules for each vaccine consisted of either three doses over 6 months, two doses given 6 months apart, or a single dose.

### Procedures

At enrollment, participants were asked to provide two nurse-assisted self-administered vaginal swabs that were used for HPV testing and genotyping with the Anyplex II HPV 28 detection assay (Seegene, Seoul, Korea) at the Catalan Institute of Oncology, Barcelona^24^. For this study, antibody responses to the nine HPV genotypes (HPV 6, 11, 16, 18, 31, 33, 45, 52, and 58) included in 9vHPV were evaluated utilizing a Luminex bead-based multiplex immunoassay. A random sample of 75 girls per arm was selected from the one and two dose 9vHPV arms for HPV multiplex immunoassay testing at month (M)0, M7, M12, M24, and M36. Only the one and two dose arms were evaluated, to coincide with the current label indications for 9vHPV vaccine (two doses of HPV vaccine for 9–14 year old participants who are immunocompetent) and the off-label (single dose of HPV vaccine for 9–20 year old participants who are immunocompetent) recommendation from WHO. The Mwanza National Institute for Medical Research laboratory processed and froze the serum samples at –80 °C and shipped them to the Frederick National Laboratory for Cancer Research’s HPV Serology Laboratory (HPVSL) in the United States for immunoassay testing. HPVSL was blinded to the sample’s status (vaccine type and dose).

### HPV multiplex luminex-based immunoassay (LIA9v)

Briefly, HPV virus-like particles (VLPs) were produced using a previously described protocol for HPV 6, 11, 16, 18, 31, 33, 45, 52, and 58 with slight modifications^25,26^. Human Embryonic Kidney 293TT cells were transfected with type-specific HPVpSheLL plasmids encoding L1 and L2 proteins for all types except HPV 11 and HPV 33 VLPs, for which pVITRO11L1L2 and pVITRO33L1L2 plasmids were used, respectively. Cell lysates were incubated after transfection at 37 °C for 24 h, followed by purification through Optiprep™ (Sigma-Aldrich, St. Louis, MO) ultracentrifugation. Fractions were collected and evaluated for HPV protein concentration and purity. Acceptable fractions were pooled, and VLP qualification and storage were performed.

HPV 6, 11, 16, 18, 31, 33, 45, 52, and 58 serum IgG antibody levels were measured by a VLP-based multiplex immunoassay at the HPVSL. In brief, anti-HPV IgG antibodies were detected by a multiplex immunoassay, with HPV 6, 11, 16, 18, 31, 33, 45, 52, and 58 L1L2 VLPs coupled to unique sets of Luminex magnetic beads. Initially, serum samples from participants and controls were serially diluted threefold in Dulbecco’s phosphate-buffered saline (DPBS) and 1% bovine serum albumin (BSA) (diluent), starting at 1:50 and ending at 1:1350. Standard was serially diluted eight times in diluent in three-fold increments. Serial dilutions of samples and control specimens were plated in singlet, and the standard was plated in duplicate in a black 96-well flat bottom non-binding plate. Next, the HPV 6, 11, 16, 18, 31, 33, 45, 52, and 58 L1L2 VLPs coupled to unique sets of Luminex magnetic beads were added to the 96-well plate to incubate with the samples, controls, and standard at room temperature for 30 min, shaking at 750 RPM. Following incubation, the 96-well plate was washed, and then goat anti-human IgG R-phycoerythrin conjugate was added to the plate. The 96-well plate was incubated at room temperature for 30 minutes, shaking at 750 RPM. The 96-well plate was washed again, and buffer (DPBS and 0.05% Tween 20) was added to each well. The plate was incubated at room temperature for ≥10 min, shaking at 750 RPM prior to reading on the Luminex instrument. Sample anti-HPV 6, 11, 16, 18, 31, 33, 45, 52, and 58 IgG antibody levels were calculated by interpolation of Median Fluorescent Intensity (MFI) values from the standard curve by averaging the calculated concentrations from all dilutions that fell within the working range of the standard curve. We utilized the 7 newly developed and approved HPV antibody WHO International Standards (anti-HPV 6, HPV 11, HPV 31, HPV 33, HPV 45, HPV 52, and HPV 58)^27^ along with the previously approved anti-HPV 16 and HPV 18 antibody WHO International Standards to calibrate our HPV multiplex immunoassay, so results are presented in International Units per milliliter (IU/mL). The seropositive cutoff value set for our assay was developed from a two-step approach. The first step included the testing of children’s serum (presumable unexposed to HPV) and calculated the upper 95% confidence interval (CI) of the geometric mean of distribution. In the second step, we utilized serum from HPV vaccinated participants, whose sample was serially diluted until the variability reached a percent error ≤50% and relative standard deviation (RSD) ≤ 30%. The highest value obtained from these two steps was utilized to set the cutoff value. The seropositive cutoff for each HPV genotype was as follows: HPV 6, 7.5 IU/mL; HPV 11, 2.7 IU/mL; HPV 16, 0.9 IU/mL; HPV 18, 0.5 IU/mL; HPV 31, 1.5 IU/mL; HPV 33, 1.8 IU/mL; HPV 45, 3.0 IU/mL; HPV 52, 12.9 IU/mL; HPV 58, 6.2 IU/mL.

### Statistical analysis

The primary analysis was conducted in the per-protocol cohort, defined as girls who received the allocated doses of vaccine within the protocol-defined window and who were HPV antibody and DNA negative at enrollment for the specific genotype under analysis. As a sensitivity analysis, the analyses were repeated in all participants who received at least one dose of HPV vaccine, irrespective of their baseline antibody or HPV DNA status.

The number and proportion of girls who were seropositive for each of the 9vHPV vaccine genotype-specific antibodies were tabulated at M24 and M36, the timepoints by when single-dose antibody concentrations are expected to have reached plateau levels^5^. For each vaccine type and HPV genotype, the difference (one-dose minus two-dose) in the proportion seropositive and the 95% CI for the difference were calculated using the exact method described by Chan and Zhang^28^.

For the evaluation of antibody geometric mean concentrations (GMC), HPV genotype-specific antibody concentrations were first log10-transformed; those below the assay cut-off were given a value of half the cut-off before log transformation. The arithmetic mean log10 antibody concentration and 95% CIs were calculated for each arm, assuming a normal distribution.

Stability of the immune responses was assessed by estimating the fold change in each of the 9vHPV vaccine genotype-specific antibody GMCs between M36 and the earlier time points (M24 and M12). For each HPV genotype and vaccine type, a linear mixed effects model with log10 antibody concentration was fitted as the response variable, dose group, time point, and a dose group-time interaction term as fixed effects, and participant as a random effect to account for correlation of repeated measurements within participants. The change over time in genotype-specific log10 concentrations (e.g. M36 minus M24) was estimated from this model, and the GMC ratio (M36/M24) and its 95% CI were obtained by back-transformation.

## Supplementary information

Supplementary Information

## Data Availability

De-identified participant data presented in this manuscript can be made available after publication following written request to the London School of Hygiene & Tropical Medicine and the Mwanza Intervention Trials Unit, Tanzania. Requests must be accompanied by a defined analysis plan addressed to the corresponding author which will be reviewed by the Mwanza Intervention Trials Unit Data Sharing Committee and senior investigators at the London School of Hygiene & Tropical Medicine. Requesting researchers will be required to sign a Data Access Agreement if approval is given.
