# Supplementary material for: Immunogenicity of one and two doses of Gardasil®9 in Tanzanian girls in the DoRIS Trial
Source: NPJ Vaccines. 2025 Dec 19;11:22. doi: 10.1038/s41541-025-01344-1 (PMC12827318; doi:10.1038/s41541-025-01344-1)
Supplement: Supplementary file 1 — Supplementary Information [file 41541_2025_1344_MOESM1_ESM.pdf]

Supplementary Table 1. Comparisons of antibody seropositivity at M7 to M36 after one- or two-doses of Gardasil®9 in DoRIS trial (total vaccinated cohort[1])

	One-dose		Two-doses		Difference in seropositivity[2] (exact 95% CI)
	N	Seropositive[2] (%)	N	Seropositive[2] (%)	[One-dose] – [Two-dose]
Gardasil®9					
**Month 7**					
HPV 6	75	65 (86.7%)	75	75 (100.0%)	-13.3% (-23.2, -6.5)
HPV 11	75	74 (98.7%)	75	75 (100.0%)	-1.3% (-7.3, 3.6)
HPV 16	75	75 (100.0%)	75	75 (100.0%)	0
HPV 18	75	75 (100.0%)	75	75 (100.0%)	0
HPV 31	75	74 (98.7%)	75	75 (100.0%)	-1.3% (-7.3, 3.6)
HPV 33	75	75 (100.0%)	75	75 (100.0%)	0
HPV 45	75	62 (82.7%)	75	75 (100.0%)	-17.3% (-27.8, -9.5)
HPV 52	75	61 (81.3%)	75	75 (100.0%)	-18.7% (-29.4, -10.6)
HPV 58	75	74 (98.7%)	75	75 (100.0%)	-1.3% (-7.3, 3.6)
**Month 12**					
HPV 6	75	63 (84.0%)	75	75 (100.0%)	-16.0% (-26.3, -8.5)
HPV 11	75	74 (98.7%)	75	75 (100.0%)	-1.3% (-7.3, 3.6)
HPV 16	75	75 (100.0%)	75	75 (100.0%)	0
HPV 18	75	73 (97.3%)	75	75 (100.0%)	-2.7% (-9.4, 2.3)
HPV 31	75	74 (98.7%)	75	75 (100.0%)	-1.3% (-7.3, 3.6)
HPV 33	75	74 (98.7%)	75	75 (100.0%)	-1.3% (-7.3, 3.6)
HPV 45	75	61 (81.3%)	75	75 (100.0%)	-18.7% (-29.4, -10.6)
HPV 52	75	52 (69.3%)	75	75 (100.0%)	-30.7% (-42.4, -20.5)
HPV 58	75	73 (97.3%)	75	75 (100.0%)	-2.7% (-9.4, 2.3)
**Month 24**					
HPV 6	75	64 (85.3%)	75	75 (100.0%)	-14.7% (-24.7, -7.5)
HPV 11	75	72 (96.0%)	75	75 (100.0%)	-4.0% (-11.3, 1.1)
HPV 16	75	75 (100.0%)	75	75 (100.0%)	0
HPV 18	75	72 (96.0%)	75	75 (100.0%)	-4.0% (-11.3, 1.1)
HPV 31	75	75 (100.0%)	75	75 (100.0%)	0
HPV 33	75	74 (98.7%)	75	75 (100.0%)	-1.3% (-7.3, 3.6)
HPV 45	75	65 (86.7%)	75	74 (98.7%)	-12.0% (-21.8, -3.9)
HPV 52	75	53 (70.7%)	75	75 (100.0%)	-29.3% (-41.0, -19.4)
HPV 58	75	75 (100.0%)	75	75 (100.0%)	0
**Month 36**					
HPV 6	75	64 (85.3%)	75	75 (100.0%)	-14.7% (-24.7, -7.5)
HPV 11	75	74 (98.7%)	75	75 (100.0%)	-1.3% (-7.3, 3.6)
HPV 16	75	75 (100.0%)	75	75 (100.0%)	0
HPV 18	75	73 (97.3%)	75	75 (100.0%)	-2.7% (-9.4, 2.3)
HPV 31	75	75 (100.0%)	75	75 (100.0%)	0
HPV 33	75	73 (97.3%)	75	75 (100.0%)	-2.7% (-9.4, 2.3)
HPV 45	75	66 (88.0%)	75	74 (98.7%)	-10.7% (-20.4, -2.8)

HPV 52	75	55 (73.3%)	75	74 (98.7%)	-25.3% (-37.1, -14.9)
HPV 58	75	75 (100.0%)	75	75 (100.0%)	0

[1]DoRIS participants who received at least one dose of vaccine, irrespective of their HPV DNA or serostatus at baseline.

[2]Seropositivity defined as antibody concentrations above the laboratory determined cut-off.

Supplementary Table 2. Stability of geometric mean concentrations (GMC) between M7 and M36 in DoRIS trial (total vaccinated cohort[1])

	One-dose		Two-doses		GMC ratio[3] (Two-doses/One-dose) 95% CI
	N[1]	GMC[2] (95% CI) (IU/mL)	N[1]	GMC[2] (95% CI) (IU/mL)	
**Gardasil®9**					
**HPV 6**					
Month 7	75	18.6 (15.1, 22.8)	75	1412.6 (1199.2,1664.1)	75.9 (57.8, 99.7)
Month 12	75	17.1 (13.9, 21.1)	75	226.7 (191.1, 268.8)	13.3 (10.1, 17.4)
Month 24	75	19.8 (15.9, 24.7)	75	122.0 (101.0, 147.5)	6.2 (4.7, 8.1)
Month 36	75	19.7 (15.9, 24.4)	75	86.3 (71.7, 104.0)	4.4 (3.3, 5.8)
GMC ratio[3] (M36 /M7) (95% CI)		*1.06 (0.95, 1.18)*		*0.06 (0.05, 0.07)*	
GMC ratio[3] (M36 /M12) (95% CI)		*1.15 (1.03, 1.29)*		*0.38 (0.34, 0.43)*	
GMC ratio[3] (M36 /M24) (95% CI)		*0.99 (0.89, 1.11)*		*0.71 (0.63, 0.79)*	
**HPV 11**					
Month 7	75	21.0 (17.8, 24.9)	75	2163.8 (1847.7,2534.0)	102.9 (79.3, 133.6)
Month 12	75	18.9 (15.6, 22.9)	75	339.6 (287.9, 400.7)	18.0 (13.9, 23.4)
Month 24	75	22.6 (18.0, 28.5)	75	175.3 (146.4, 209.9)	7.7 (6.0, 10.1)
Month 36	75	23.2 (18.8, 28.8)	75	121.1 (100.8, 145.3)	5.2 (4.0, 6.8)
GMC ratio[3] (M36 /M7) (95% CI)		*1.11 (0.98, 1.24)*		*0.06 (0.05, 0.06)*	
GMC ratio[3] (M36 /M12) (95% CI)		*1.23 (1.10, 1.38)*		*0.36 (0.32, 0.40)*	
GMC ratio[3] (M36 /M24) (95% CI)		*1.03 (0.91, 1.15)*		*0.69 (0.61, 0.78)*	
**HPV 16**					
Month 7	75	11.3 (9.2, 13.9)	75	1293.3 (1106.7,1511.3)	114.3 (86.4, 151.2)
Month 12	75	11.1 (8.8, 14.0)	75	250.4 (210.4, 297.9)	22.5 (17.0, 29.8)
Month 24	75	14.4 (11.5, 18.1)	75	124.7 (103.0, 151.0)	8.6 (6.5, 11.4)
Month 36	75	13.2 (10.6, 16.4)	75	88.5 (72.6, 107.8)	6.7 (5.1, 8.9)
GMC ratio[3] (M36 /M7) (95% CI)		*1.17 (1.03, 1.31)*		*0.07 (0.06, 0.08)*	
GMC ratio[3] (M36 /M12) (95% CI)		*1.19 (1.05, 1.34)*		*0.35 (0.31, 0.40)*	
GMC ratio[3] (M36 /M24) (95% CI)		*0.91 (0.81, 1.03)*		*0.71 (0.63, 0.80)*	
**HPV 18**					
Month 7	75	3.8 (3.2, 4.5)	75	326.1 (267.8, 397.1)	85.6 (62.5, 117.4)
Month 12	75	3.5 (2.8, 4.4)	75	49.6 (39.4, 62.5)	14.3 (10.4, 19.6)
Month 24	75	4.1 (3.2, 5.1)	75	24.1 (18.7, 31.1)	5.9 (4.3, 8.1)
Month 36	75	4.0 (3.2, 5.1)	75	17.1 (13.1, 22.3)	4.2 (3.1, 5.8)
GMC ratio[3] (M36 /M7) (95% CI)		*1.06 (0.94, 1.20)*		*0.05 (0.05, 0.06)*	
GMC ratio[3] (M36 /M12) (95% CI)		*1.16 (1.03, 1.32)*		*0.34 (0.30, 0.39)*	
GMC ratio[3] (M36 /M24) (95% CI)		*0.99 (0.88, 1.13)*		*0.71 (0.63, 0.80)*	
**HPV 31**					
Month 7	75	15.0 (12.3, 18.2)	75	1386.0 (1184.6,1621.7)	92.6 (70.5, 121.5)
Month 12	75	14.9 (12.1, 18.4)	75	233.5 (196.4, 277.7)	15.6 (11.9, 20.5)
Month 24	75	19.7 (16.0, 24.2)	75	126.6 (104.3, 153.7)	6.4 (4.9, 8.4)
Month 36	75	21.2 (16.9, 26.5)	75	93.2 (76.4, 113.6)	4.4 (3.4, 5.8)
GMC ratio[3] (M36 /M7) (95% CI)		*1.41 (1.24, 1.62)*		*0.07 (0.06, 0.08)*	
GMC ratio[3] (M36 /M12) (95% CI)		*1.42 (1.24, 1.63)*		*0.40 (0.35, 0.46)*	

GMC ratio[3] (M36 /M24) (95% CI)		*1.08 (0.94, 1.23)*		*0.74 (0.64, 0.84)*	
**HPV 33**					
Month 7	75	18.9 (15.9, 22.5)	75	1277.1 (1080.3,1509.7)	67.5 (50.9, 89.6)
Month 12	75	15.7 (12.7, 19.4)	75	215.0 (178.5, 258.8)	13.7 (10.3, 18.2)
Month 24	75	19.0 (15.1, 23.9)	75	114.9 (94.1, 140.2)	6.1 (4.6, 8.0)
Month 36	75	21.0 (16.5, 26.6)	75	81.1 (66.0, 99.6)	3.9 (2.9, 5.1)
GMC ratio[3] (M36 /M7) (95% CI)		*1.11 (0.96, 1.28)*		*0.06 (0.06, 0.07)*	
GMC ratio[3] (M36 /M12) (95% CI)		*1.34 (1.16, 1.55)*		*0.38 (0.33, 0.44)*	
GMC ratio[3] (M36 /M24) (95% CI)		*1.11 (0.96, 1.28)*		*0.71 (0.61, 0.81)*	
**HPV 45**					
Month 7	75	6.5 (5.3, 8.0)	75	601.2 (505.8, 714.6)	92.3 (69.3, 122.8)
Month 12	75	6.4 (5.2, 7.8)	75	89.4 (73.5, 108.7)	14.0 (10.5, 18.7)
Month 24	75	7.8 (6.3, 9.7)	75	46.0 (37.0, 57.2)	5.9 (4.4, 7.8)
Month 36	75	7.9 (6.4, 9.8)	75	35.0 (28.3, 43.3)	4.4 (3.3, 5.9)
GMC ratio[3] (M36 /M7) (95% CI)		*1.21 (1.08, 1.36)*		*0.06 (0.05, 0.07)*	
GMC ratio[3] (M36 /M12) (95% CI)		*1.24 (1.10, 1.39)*		*0.39 (0.35, 0.44)*	
GMC ratio[3] (M36 /M24) (95% CI)		*1.01 (0.89, 1.13)*		*0.76 (0.68, 0.86)*	
**HPV 52**					
Month 7	75	24.5 (20.4, 29.4)	75	1534.3 (1312.7,1793.2)	62.7 (48.0, 81.8)
Month 12	75	19.5 (16.1, 23.7)	75	297.7 (249.2, 355.7)	15.3 (11.7, 19.9)
Month 24	75	22.2 (18.0, 27.5)	75	155.0 (127.7, 188.1)	7.0 (5.3, 9.1)
Month 36	75	23.6 (19.1, 29.1)	75	109.2 (89.4, 133.6)	4.6 (3.6, 6.0)
GMC ratio[3] (M36 /M7) (95% CI)		*0.96 (0.86, 1.08)*		*0.07 (0.06, 0.08)*	
GMC ratio[3] (M36 /M12) (95% CI)		*1.21 (1.08, 1.36)*		*0.37 (0.33, 0.41)*	
GMC ratio[3] (M36 /M24) (95% CI)		*1.06 (0.94, 1.19)*		*0.71 (0.63, 0.79)*	
**HPV 58**					
Month 7	75	61.2 (50.9, 73.5)	75	3713.2 (3199.0,4310.0)	60.7 (46.8, 78.8)
Month 12	75	58.2 (46.4, 72.9)	75	730.7 (621.7, 858.8)	12.6 (9.7, 16.3)
Month 24	75	87.9 (71.0, 108.7)	75	393.6 (332.3, 466.3)	4.5 (3.5, 5.8)
Month 36	75	95.7 (78.2, 117.1)	75	291.3 (243.2, 348.8)	3.0 (2.3, 3.9)
GMC ratio[3] (M36 /M7) (95% CI)		*1.56 (1.37, 1.78)*		*0.08 (0.07, 0.09)*	
GMC ratio[3] (M36 /M12) (95% CI)		*1.64 (1.44, 1.88)*		*0.40 (0.35, 0.45)*	
GMC ratio[3] (M36 /M24) (95% CI)		*1.09 (0.96, 1.24)*		*0.74 (0.65, 0.84)*	

[1]DoRIS participants who received at least one dose of vaccine, irrespective of their HPV DNA or serostatus at baseline. [2]Serum antibody geometric mean concentration (GMC). [3]Estimated with linear mixed effect model with log antibody concentration as the response and dose group, time point, and a dose group, time interaction term as fixed effects, and participant as a random effect to account for correlation of repeated measurements within participant.

Supplementary Figure:

1a.

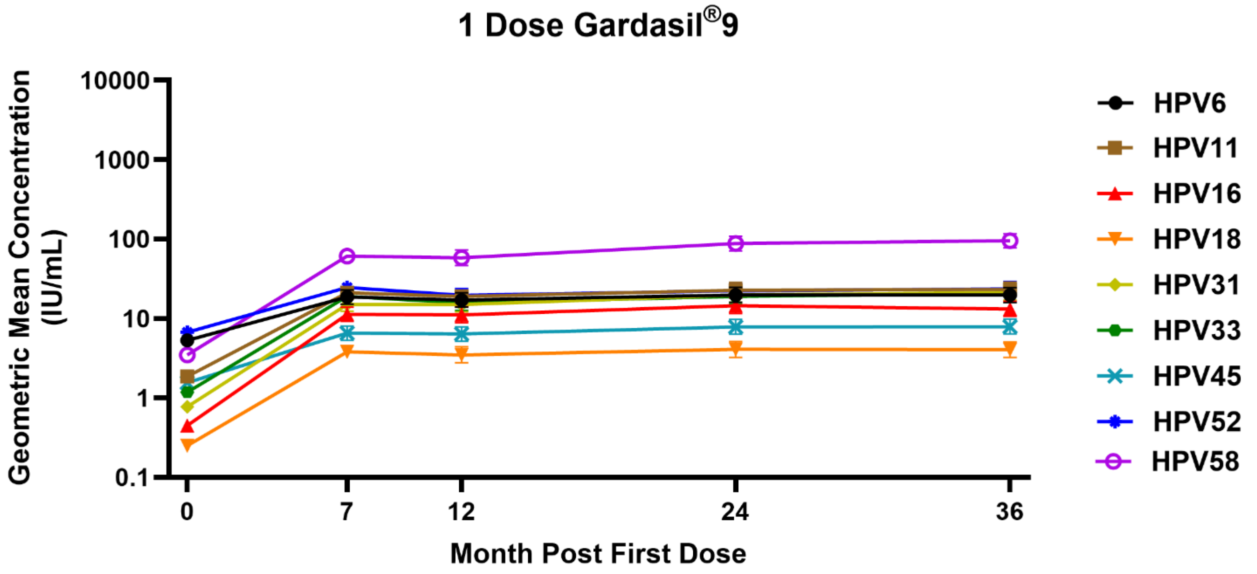

1b.

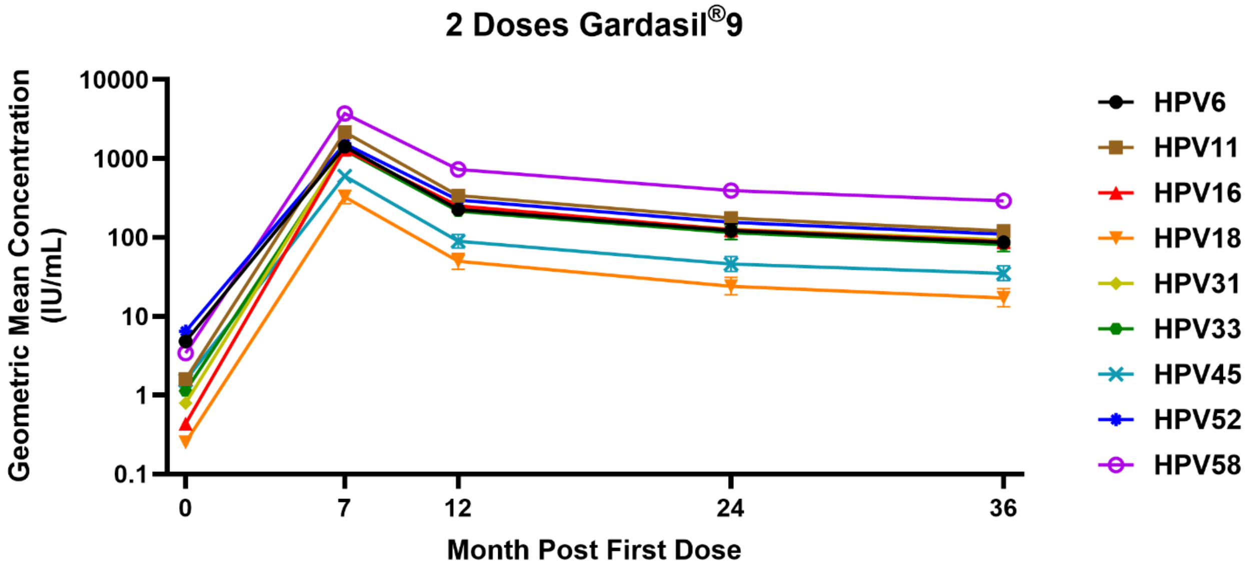

Supplementary Figure 1. Stability of HPV IgG antibody levels up to Month (M)36 in the total vaccinated cohort amongst one- and two-dose recipients of the 9vHPV vaccine. A) depicts the geometric mean concentration in International Units (IU) per milliliter (mL) anti-HPV IgG antibody responses for the nine HPV genotypes included in the 9vHPV vaccine for one-dose recipients of the vaccine, while B) depicts the geometric mean concentration in IU/mL anti-HPV IgG antibody responses for the nine HPV genotypes included in the 9vHPV vaccine for two-dose recipients of the vaccine. The x-axis describes the timepoints (M0, M7, M12, M24, and M36) of sample testing, and the y-axis indicates the measured concentration in IU/mL. The symbols indicate the geometric mean concentration (IU/mL) for each HPV genotype evaluated, and the error bars associated with each symbol indicate the 95% confidence interval.

Section/topic	No	CONSORT 2025 checklist item description	Reported on page no.
**Title and abstract**			
Title and structured abstract	1a	Identification as a randomised trial	2
	1b	Structured summary of the trial design, methods, results, and conclusions	2- structured per Journal specs
**Open science**			
Trial registration	2	Name of trial registry, identifying number (with URL) and date of registration	2
Protocol and statistical analysis plan	3	Where the trial protocol and statistical analysis plan can be accessed	11-Reference#23
Data sharing	4	Where and how the individual de-identified participant data (including data dictionary), statistical code and any other materials can be accessed	15
Funding and conflicts of interest	5a	Sources of funding and other support (eg, supply of drugs), and role of funders in the design, conduct, analysis and reporting of the trial	15
	5b	Financial and other conflicts of interest of the manuscript authors	16
**Introduction**			
Background and rationale	6	Scientific background and rationale	3
Objectives	7	Specific objectives related to benefits and harms	3
**Methods**			
Patient and public involvement	8	Details of patient or public involvement in the design, conduct and reporting of the trial	11
Trial design	9	Description of trial design including type of trial (eg, parallel group, crossover), allocation ratio, and framework (eg, superiority, equivalence, non-inferiority, exploratory)	11
Changes to trial protocol	10	Important changes to the trial after it commenced including any outcomes or analyses that were not prespecified, with reason	11-Reference#23
Trial setting	11	Settings (eg, community, hospital) and locations (eg, countries, sites) where the trial was conducted	11
Eligibility criteria	12a	Eligibility criteria for participants	11-Reference#23
	12b	If applicable, eligibility criteria for sites and for individuals delivering the interventions (eg, surgeons, physiotherapists)	11-Reference#23
Intervention and comparator	13	Intervention and comparator with sufficient details to allow replication. If relevant, where additional materials describing the intervention and comparator (eg, intervention manual) can be accessed	11
Outcomes	14	Prespecified primary and secondary outcomes, including the specific measurement variable (eg, systolic blood pressure), analysis metric (eg, change from baseline, final value, time to event), method of aggregation (eg, median, proportion), and time point for each outcome	11
Harms	15	How harms were defined and assessed (eg, systematically, non-systematically)	N/A to current study, but can be assessed at

			11-Reference#23
Sample size	16a	How sample size was determined, including all assumptions supporting the sample size calculation	11
	16b	Explanation of any interim analyses and stopping guidelines	11, 13-14
Randomisation:			
Sequence generation	17a	Who generated the random allocation sequence and the method used	Dr. Kathy Baisley
	17b	Type of randomisation and details of any restriction (eg, stratification, blocking and block size)	11, 13-14
			**Reported on page no.**
Allocation concealment mechanism	18	Mechanism used to implement the random allocation sequence (eg, central computer/telephone; sequentially numbered, opaque, sealed containers), describing any steps to conceal the sequence until interventions were assigned	11, 13-14
Implementation	19	Whether the personnel who enrolled and those who assigned participants to the interventions had access to the random allocation sequence	11-Reference#23
Blinding	20a	Who was blinded after assignment to interventions (eg, participants, care providers, outcome assessors, data analysts)	11-12, and Reference#23
	20b	If blinded, how blinding was achieved and description of the similarity of interventions	11-12, and Reference#23
Statistical methods	21a	Statistical methods used to compare groups for primary and secondary outcomes, including harms	13-14
	21b	Definition of who is included in each analysis (eg, all randomised participants), and in which group	11
	21c	How missing data were handled in the analysis	11-12, 13-14
	21d	Methods for any additional analyses (eg, subgroup and sensitivity analyses), distinguishing prespecified from post hoc	13-14
**Results**			
Participant flow, including flow diagram	22a	For each group, the numbers of participants who were randomly assigned, received intended intervention, and were analysed for the primary outcome	5, and Table 1
	22b	For each group, losses and exclusions after randomisation, together with reasons	5, and Table 1
Recruitment	23a	Dates defining the periods of recruitment and follow-up for outcomes of benefits and harms	11-Reference#23
	23b	If relevant, why the trial ended or was stopped	N/A
Intervention and comparator delivery	24a	Intervention and comparator as they were actually administered (eg, where appropriate, who delivered the intervention/comparator, how participants adhered, whether they were delivered as intended (fidelity))	5, and Table 1, 11-Reference#23
	24b	Concomitant care received during the trial for each group	11-Reference#23
Baseline data	25	A table showing baseline demographic and clinical characteristics for each group	11-Reference#23, and Table 1
Numbers analysed, outcomes and estimation	26	For each primary and secondary outcome, by group:<br>● the number of participants included in the analysis<br>● the number of participants with available data at the outcome time point	5-6, Table 1, 2, 3

		● result for each group, and the estimated effect size and its precision (such as 95% confidence interval)<br>● for binary outcomes, presentation of both absolute and relative effect size	
Harms	27	All harms or unintended events in each group	11-Reference#23
Ancillary analyses	28	Any other analyses performed, including subgroup and sensitivity analyses, distinguishing pre-specified from post hoc	5-6
**Discussion**			
Interpretation	29	Interpretation consistent with results, balancing benefits and harms, and considering other relevant evidence	7-9
Limitations	30	Trial limitations, addressing sources of potential bias, imprecision, generalisability, and, if relevant, multiplicity of analyses	9-10

Citation: Hopewell S, Chan AW, Collins GS, Hróbjartsson A, Moher D, Schulz KF, et al. CONSORT 2025 Statement: updated guideline for reporting randomised trials. BMJ. 2025; 388:e081123.
https://dx.doi.org/10.1136/bmj-2024-081123
© 2025 Hopewell et al. This is an Open Access article distributed under the terms of the Creative Commons Attribution License (https://creativecommons.org/licenses/by/4.0/), which permits unrestricted use, distribution, and reproduction in any medium, provided the original work is properly cited.

*We strongly recommend reading this statement in conjunction with the CONSORT 2025 Explanation and Elaboration and/or the CONSORT 2025 Expanded Checklist for important clarifications on all the items. We also recommend reading relevant CONSORT extensions. See www.consort-spirit.org.
